# Association between bone marrow lesions and bone mineral density of the proximal tibia in end-stage osteoarthritic knees

**DOI:** 10.1038/s41598-023-33251-7

**Published:** 2023-04-21

**Authors:** Eiji Sasaki, Ryo Araki, Tomoyuki Sasaki, Yuji Wakai, Yuji Yamamoto, Yasuyuki Ishibashi

**Affiliations:** 1 Department of Orthopaedic Surgery, Hirosaki University Graduate School of Medicine, 5 Zaifu-Cho, Hirosaki, Aomori 036-8562 Japan; 2 Department of Orthopaedic Surgery, Hirosaki Memorial Hospital, Hirosaki, Japan

**Keywords:** Biomarkers, Rheumatology

## Abstract

This retrospective cross-sectional study investigated the association between bone marrow lesions (BMLs) and bone mineral density (BMD) in the proximal tibia of end-stage osteoarthritic knees from a large patient sample. Overall, 1308 end-stage osteoarthritic knees were enrolled before total knee arthroplasty. The preoperative range of motion was recorded. Bone mineral density in the medial tibial plateau (MTP), lateral tibial plateau (LTP), and metaphysis were measured using dual-energy X-ray absorptiometry. The MTP/LTP, MTP/metaphysis, and LTP/metaphysis ratios were calculated. BMLs were scored using a whole-organ magnetic resonance imaging scoring system. The relationship between BMD and BML scores was investigated using linear regression analysis. The highest BMD was 0.787 ± 0.176 g/cm^2^ at the MTP, followed by 0.676 ± 0.180 g/cm^2^ and 0.572 ± 0.145 g/cm^2^ at the metaphysis and LTP, respectively. The prevalence of BMLs was 90.4% and 24.2% in the MTP and LTP, respectively. In women, higher BML scores at the MTP were positively correlated with the BMD of the MTP (p < 0.001, r = 0.278), MTP/LTP (p < 0.001, r = 0.267), and MTP/metaphysis ratios (p < 0.001, r = 0.243). Regression analysis showed that higher BML scores in the MTP were correlated with higher BMD in the MTP (p < 0.001) and lower BMD in the LTP (p < 0.001). High BML scores in the MTP were positively associated with high BMD in the MTP, which also induced the medial to lateral imbalance of BMD in the proximal tibia.

## Introduction

Sufficient bone strength is essential for successful total knee arthroplasty (TKA). Despite its promising long-term clinical outcomes^1,2^, complications such as aseptic loosening and periprosthetic fractures have been reported in patients^3,4^. Because lower preoperative bone mineral density (BMD) of the tibial plateau is associated with an increased risk of tibial component migration after TKA^5^, bone fragility and lower BMD must be addressed preoperatively. However, unlike the lumbar spine and proximal femur, BMD measurement of the proximal tibia is not common in clinical practice, and these measurements are not widely used. Thus, little is known about age-related changes of BMD in the proximal tibia and its effect on the incidence of complications after TKA.

Here, we focused on bone marrow lesions (BMLs) on magnetic resonance imaging (MRI) as a possible indicator for focal bone strength in the proximal tibia. BML, a common finding detected on MRI during the early- to end-stage of knee osteoarthritis (OA)^6–8^, which is associated with knee pain^9,10^, predicts radiographic structural changes^11,12^ and indicates the need for joint replacement^13^. BML histology reveals microcracks, bone remodeling, edema, fibrosis, and bleeding in the subchondral bone^14,15^; hence, BMLs may indicate possible weakness of the subchondral bone. An epidemiological study showed that BMD in patients with early knee OA was lower, with a high turnover of bone metabolism^16^. However, the relationship between BMD and BML in the proximal tibia of patients with end-stage knee OA remains unclear. These relationships with BMLs may be important to consider, without the need for an additional perioperative intervention or surgical planning, given that MRI scanning has become a routine preoperative examination.

This cross-sectional study aimed to examine BMD of the proximal tibia in patients with end-stage knee OA for age-related changes. Additionally, the association between BMLs and BMD in the proximal tibia was investigated in a large patient population. We hypothesized that BML size would correlate with increased BMD due to sclerosis of the subchondral bone. Examining these relationships may aid in understanding the etiology of end-stage knee OA and clinicians’ planning for perioperative therapeutic strategies.

## Methods

### Patients

Overall, 1621 TKA procedures were performed between November 2007 and September 2018. Among consecutive patients who underwent TKA, the following were excluded from the study: (1) 12 patients with rheumatoid arthritis, (2) one patient treated for knee joint infection, (3) 12 patients who underwent postoperative high tibial osteotomy, and (4) 281 patients with incomplete datasets. Finally, data from 837 patients (1307 knees; 106 men, 731 women) were analyzed. Demographic data of all patients at the time of surgery, including age, sex, height, weight, body mass index (BMI), loss of knee extension angle, and knee flexion angle, were retrospectively collected from medical records. All patients’ data were de-identified. All participants provided written informed consent, and the study was done in agreement with the 1964 Helsinki Declaration and its later amendments or comparable ethical standards and conducted with approval by the ethics committee of the Hirosaki Memorial Hospital (No. 2021-05, approved date: Jul. 29th, 2021). The reporting of this study conforms to the STROBE statement^17^.

### BMD measurement

**Figure 1:**
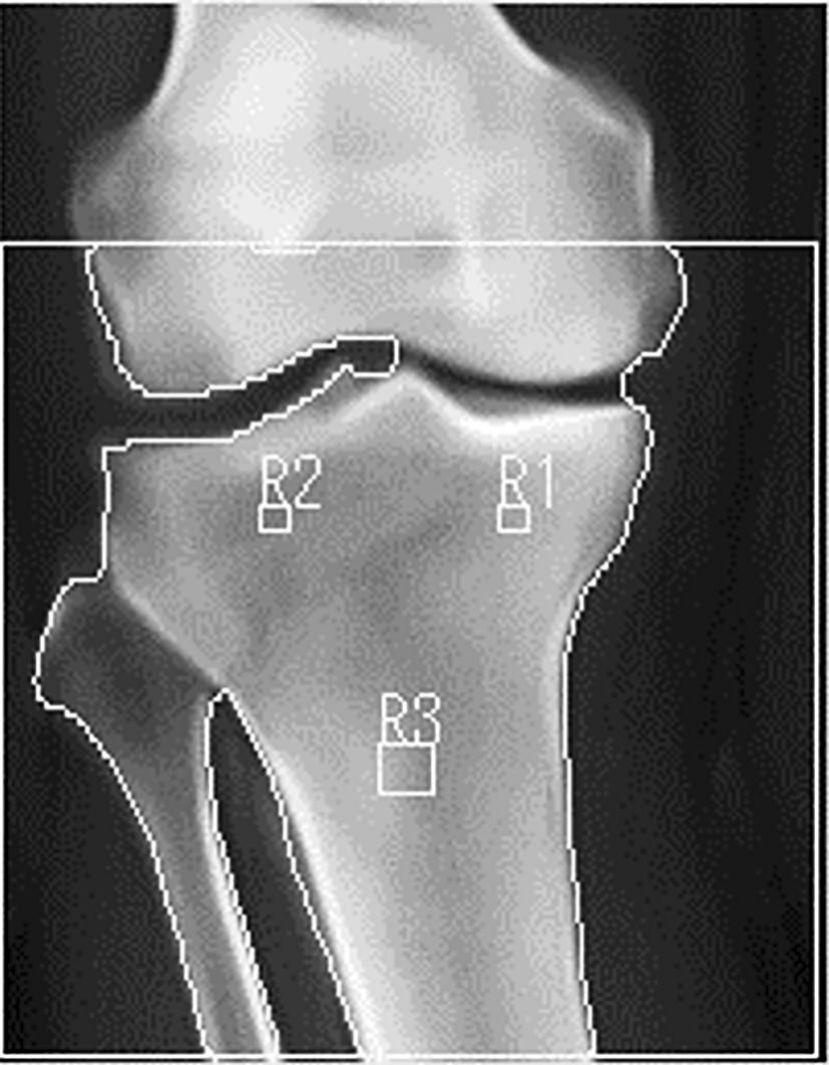
Regions of interest (ROIs) in the proximal tibia for evaluating bone mineral density. ROI of the medial tibial plateau (R1): center of the medial tibial plateau 10 mm distal to the subchondral bone on the anterior–posterior scout image. ROI of the lateral tibial plateau (R2): the center of the lateral tibial plateau, 10 mm distal to the subchondral bone on the anterior–posterior scout image. ROI of the metaphysis (R3): the center of the tibia, 50 mm distal to the joint line of the proximal tibia.

BMD of the proximal tibia was determined by dual-energy X-ray absorptiometry using Horizon W (S/N200827, Hologic, Inc. Tokyo, Japan). Patients were positioned supine with their knee flexed at 10° using a customized knee and ankle joint supportive device that positioned the knee with the patella facing upward. BMD was measured in the metaphysis, medial, and lateral parts of the proximal tibia. The region of interest (ROI) of the medial tibia was defined as the center of the tibial plateau, 10 mm distal to the subchondral bone on the AP scout image, shown as R1 (Fig. 1). The ROI of the lateral tibia was defined in the same manner, shown as R2 in Fig. 1. The ROI of the metaphysis was defined as the center of the tibia, 50 mm distal to the joint line of the proximal tibia, shown as R3 (Fig. 1). The size of ROI was uniformly set as 0.6 cm^2^. The medial tibial plateau/lateral tibial plateau (MTP/LTP), MTP/metaphysis, and LTP/metaphysis ratios were calculated to estimate the medial to lateral balance of BMD in the proximal tibia.

### BML scores in MRI

**Figure 2:**
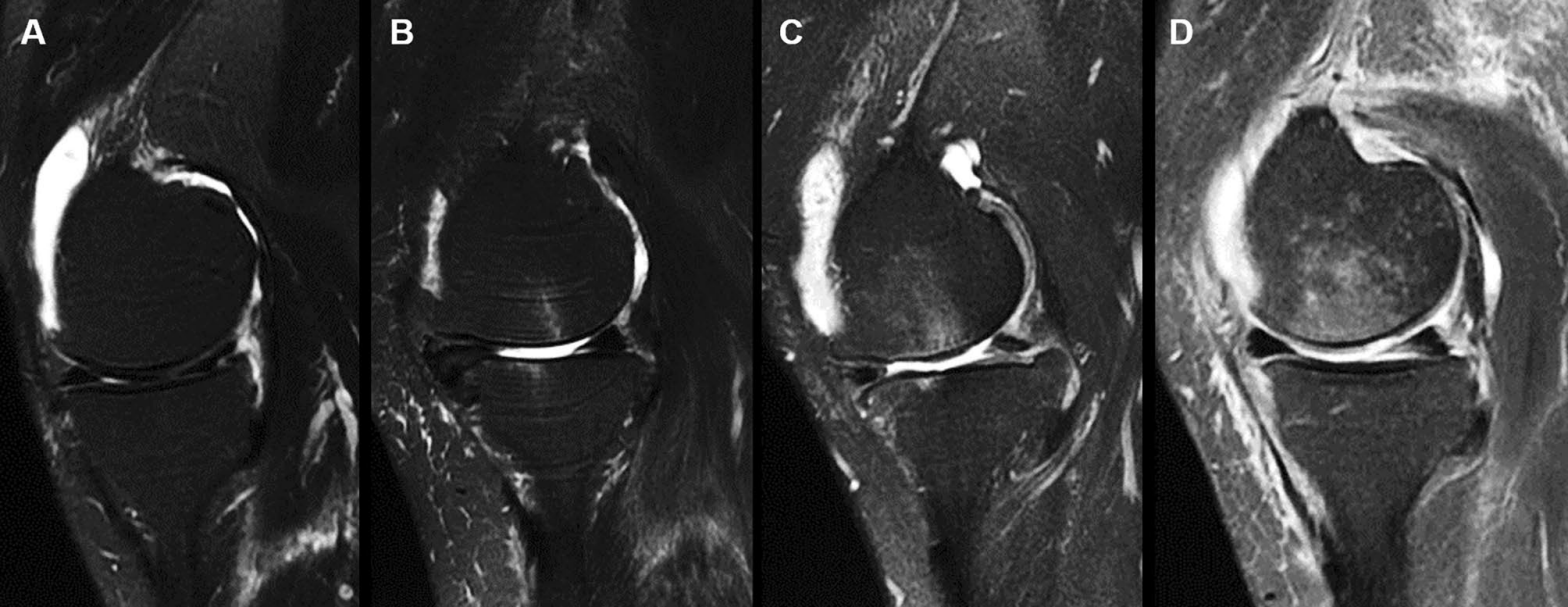
Bone marrow lesion scores using magnetic resonance imaging. Sagittal and coronal views of short TI inversion recovery (STIR) images of the knee are taken. Bone marrow lesions (BMLs) are scored using the whole magnetic resonance imaging score, where A is scored as grade 0, B as grade 1, C as grade 2, and D as grade 3 in the medial femoral condyle.

MRI scans of the involved knee were taken using a rapid extremity coil and mobile magnetic resonance unit (1.5 T; 8-channel knee phased array coil, GE Healthcare, Tokyo, Japan) preoperatively. Patients were positioned supine with their knees in full extension. Sequences included sagittal and coronal T2-weighted fat saturation-fast spin-echo (repetition time, 2400 ms; echo time, 90 ms; field of view, 10–20 cm; 320 × 224 matrix; 4-mm thick slices with a 1-mm interslice gap). BML was defined as an area of irregular hyperintense signals in the subchondral bone. The area was measured semi-quantitatively using the whole-organ MRI scoring method in 15 subregions^18^. Specifically, MTP, LTP, medial femoral condyle (MFC), and lateral femoral condyle (LFC) were divided into three subregions (anterior, central, and posterior), and the tibia had one additional subregion that represented the area below the tibial spine. The patella was divided into medial and lateral subregions. BMLs were each scored as integers from 0 to 3, where 0 = normal; 1 = mild, < 25% of the region; 2 = moderate, 25–50% of the region; and 3 = severe, > 50% of the region (Fig. 2).

The scores of the medial and lateral femorotibial (FT) joints were calculated as the total scores of BMLs in the center and posterior femur and the total scores of BMLs in three regions of the tibia, respectively. The BML score of the whole joint was calculated as the sum of the abovementioned 15 subregional scores. A BML was considered present if the total BML score was greater than zero. One blinded observer, an orthopedic surgeon, scored MR images with no access to the patients’ clinical information, and these scores were used for statistical analyses. Furthermore, to validate the scoring reliability, the BMLs of 100 randomly selected MR images were scored by an independent blinded orthopedic surgeon. The inter-rater reliability of the two observers, expressed as intraclass correlation coefficients (2.1), were 0.920 (95% confidence interval [CI] 0.884–0.945, p < 0.001) for medial FT scores, 0.942 (95% CI 0.915–0.960, p < 0.001) for lateral FT images, and 0.941 (95% CI 0.914–0.959, p < 0.001) for whole joint scores.

### Radiographic evaluation

Anteroposterior (AP) weight-bearing radiographs of the knee were obtained from all patients. FT angle (FTA) was measured before surgery as a parameter of the lower extremity alignment using the anatomical axis drawn at the center of the femoral and tibial shafts in AP weight-bearing knee radiographs to assess the medial or lateral type OA.

### Statistical analysis

To achieve 80% statistical power with an alpha of 0.05, power analysis revealed the requirement of a minimum of 144 patients for detecting any correlation between BML scores in MTP and BMD at MTP with Spearman’s correlation analysis. In the analysis, statistical power using 1307 patients was calculated as 1.000.

Quantitative data are expressed as mean ± SD. The chi-square test was used to compare differences in categorical variables. Mann–Whitney U test was used to compare differences in continuous variables between men and women, as most of these parameters were not normally distributed by Shapiro–Wilk tests. Spearman’s correlation coefficients (r) were estimated between BMD and BML scores and among BMD, BML scores and FTA. Linear regression analysis was performed with BML scores in the whole, MTP, and LTP as dependent variables. Age, sex, BMI, loss of knee extension and flexion, radiographic femorotibial angles, and BMD of the MTP, LTP, and metaphysis were the independent variables. Data input and analyses were performed using SPSS version 27.0 J (SPSS Inc., Chicago, IL, USA). Statistical significance was set at p < 0.05.

### Ethics approval and consent to participate

The study was conducted with the approval of the ethics committee of the Hirosaki Memorial Hospital (No. 2021-05, approved date: Jul. 29th 2021). All participants provided written informed consent.

## Results

**Figure 3:**
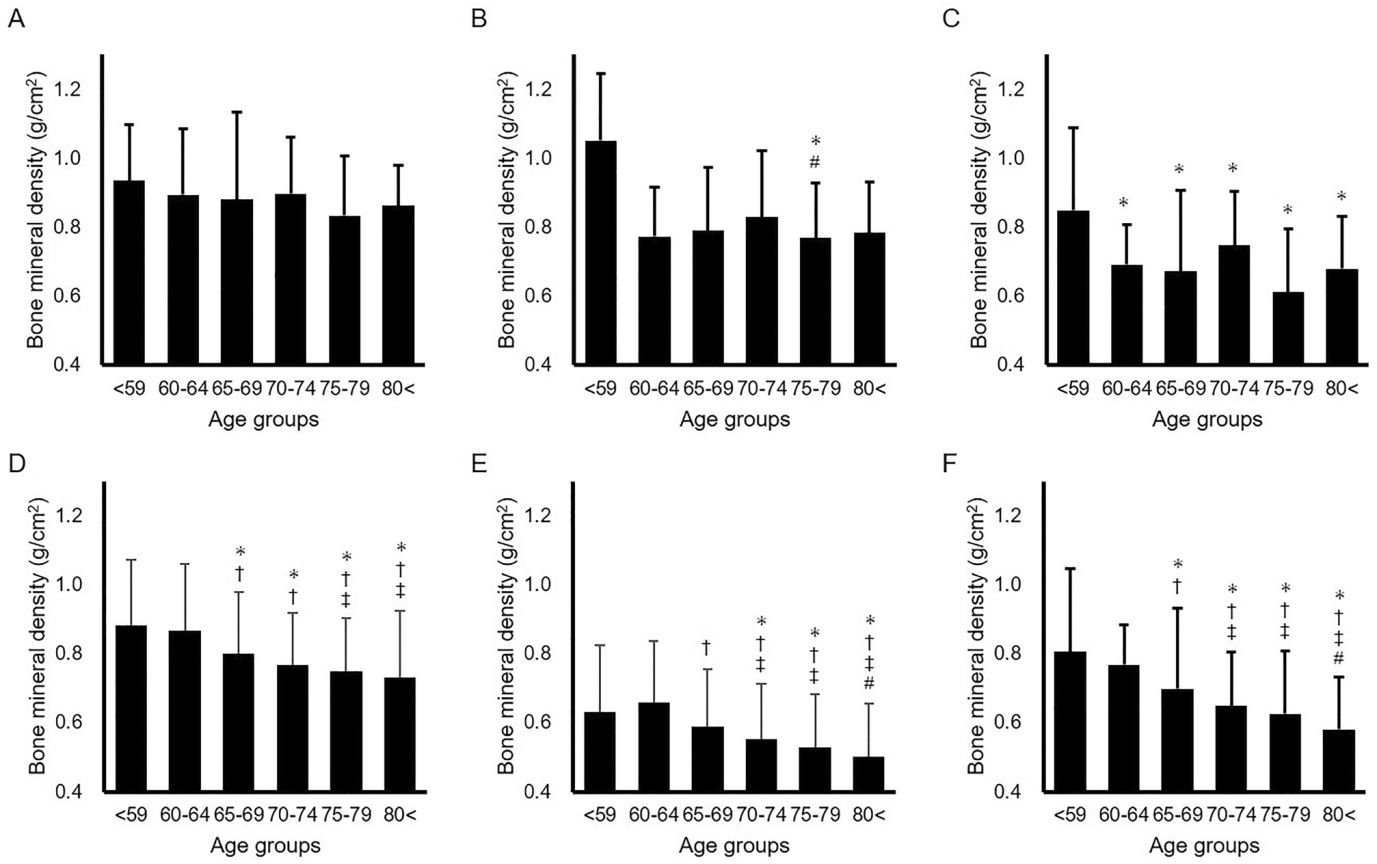
Bone mineral densities among different age groups in both sexes. Mean and standard deviations of the bone mineral density of the medial tibial plateau (**A**), lateral tibial plateau (**B**), and metaphysis (**C**) in men and those of the medial tibial plateau (**D**), lateral tibial plateau (**E**), and metaphysis (**F**) in women are shown. A p-value below 0.05 was considered significant in the comparison of the following age groups: < 59 group (*), 60–65 group (^†^), 65–69 group (^‡^), and 70–74 group (^#^).

A total of 1307 knees (from 106 men and 731 women) were enrolled for the statistical analysis. Preoperative FTAs (p < 0.001) were higher, weight and height were lower, and disease duration was longer in women than in men; however, no significance between-group differences in age, BMI, and preoperative range of motion were found (Table 1). In all patients, BMD of the MTP showed the highest value of 0.787 ± 0.176 g/cm^2^, followed by 0.676 ± 0.180 g/cm^2^ at the metaphysis and 0.572 ± 0.145 g/cm^2^ at the LTP. BMDs of the MTP, LTP, and metaphysis in women were significantly lower than those in men (Table 2). Also, the medial/lateral and medial/metaphysis ratios of women were higher than those of men, while there was no difference in the lateral/metaphysis ratio between men and women. Regarding the BMD of age groups, while fewer age-related changes were observed in men, the BMDs of the medial tibial plateau, lateral tibial plateau, and metaphysis in women decreased with age, especially in those aged > 65 years (Fig. 3).

**Table 1:** Demographics of patients who underwent primary total knee arthroplasty.

	Males	Females	p-value
Sample size	145	1162	
Age (years)	72.3 ± 7.2	72.5 ± 6.5	0.796
Disease duration (years)	10.0 ± 4.3	12.3 ± 5.0	< 0.001
Height (cm)	160.7 ± 6.7	148.7 ± 6.3	< 0.001
Weight (kg)	68.5 ± 1.1	59.6 ± 10.4	< 0.001
Body mass index (kg/m^2^)	26.4 ± 3.2	27.0 ± 6.3	0.423
Loss of knee extension angle (°)	10.8 ± 6.8	11.0 ± 8.0	0.795
Knee flexion angle (°)	124.9 ± 14.8	122.0 ± 17.8	0.052
Range of motion (°)	114.2 ± 18.3	111.1 ± 21.8	0.099
Preoperative femorotibial angle (°)	183.6 ± 5.5	185.8 ± 6.6	< 0.001

Values expressed as means ± standard deviations of continuous parameters are shown. Means ± standard deviations of males and females are compared using Mann–Whitney U test.

**Table 2:** Bone marrow lesion scores and distribution of bone mineral density of proximal tibia.

	Males		Females		p-value
	Mean	Min.–Max	Mean	Min.–Max	
Bone marrow lesion scores					
Medial femoral condyle	2.1 ± 1.5	0–6	1.9 ± 1.5	0–6	0.080
Medial tibial plateau	3.3 ± 2.4	0–9	3.5 ± 2.6	0–9	0.418
Medial femorotibial joint	5.5 ± 3.5	0–15	5.5 ± 3.8	0–15	0.743
Lateral femoral condyle	0.5 ± 1.1	0–6	0.5 ± 1.0	0–6	0.913
Lateral tibial plateau	0.5 ± 1.2	0–9	0.5 ± 1.2	0–9	0.939
Lateral femorotibial joint	1.0 ± 2.0	0–15	1.0 ± 1.9	0–14	0.864
Patellofemoral joint	1.3 ± 1.5	0–8	1.1 ± 1.3	0–10	0.145
Whole joint	8.7 ± 5.9	0–26	8.5 ± 5.8	0–38	0.817
Bone mineral density					
Medial	**0.87 ± 0.18**	**0.41–1.14**	**0.78 ± 0.17**	**0.33–1.60**	**< 0.001**
Lateral	**0.69 ± 0.18**	**0.30–1.21**	**0.56 ± 0.13**	**0.18–1.24**	**< 0.001**
Metaphysis	**0.81 ± 0.19**	**0.30–1.35**	**0.66 ± 0.17**	**0.20–1.31**	**< 0.001**
MTP/LTP ratio	**1.31 ± 0.32**	**0.46–2.55**	**1.44 ± 0.38**	**0.52–4.03**	**< 0.001**
MTP/metaphysis ratio	**1.10 ± 0.24**	**0.66–1.74**	**1.23 ± 0.32**	**0.50–3.68**	**< 0.001**
LTP/metaphysis ratio	0.88 ± 0.29	0.40–2.99	0.87 ± 0.21	0.34–2.04	0.370

Significant values are in [bold].

Means ± standard deviations of six compartments in the knee joint are shown, and values between males and females are compared using Mann–Whitney U test. Means ± standard deviations of medial, lateral, and metaphysis are shown. The medial tibial plateau/lateral tibial plateau (MTP/LTP), MTP/metaphysis, and LTP/metaphysis ratios of bone marrow density are shown, and their values are compared using Mann–Whitney U test.

**Figure 4:**
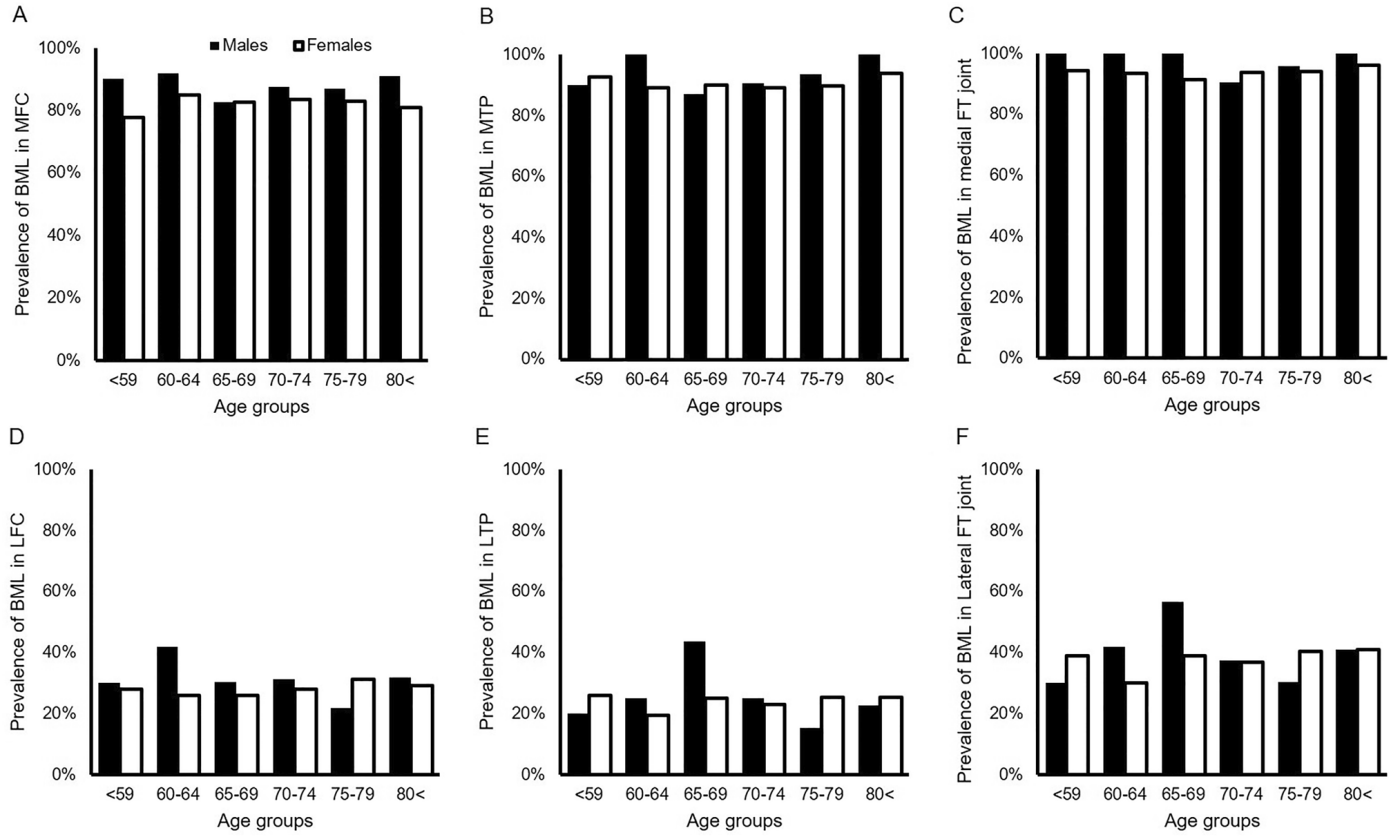
Prevalence of bone marrow lesions among different age groups in both sexes. Prevalence of bone marrow lesions (BMLs) at the medial femoral condyle (MFC) (**A**), medial tibial plateau (MTP) (**B**), and medial femorotibial (FT) joint (**C**), lateral femoral condyle (LFC) (**D**), lateral tibial plateau (LTP) (**E**), and lateral FT joint (**F**) in men and women are shown.

**Figure 5:**
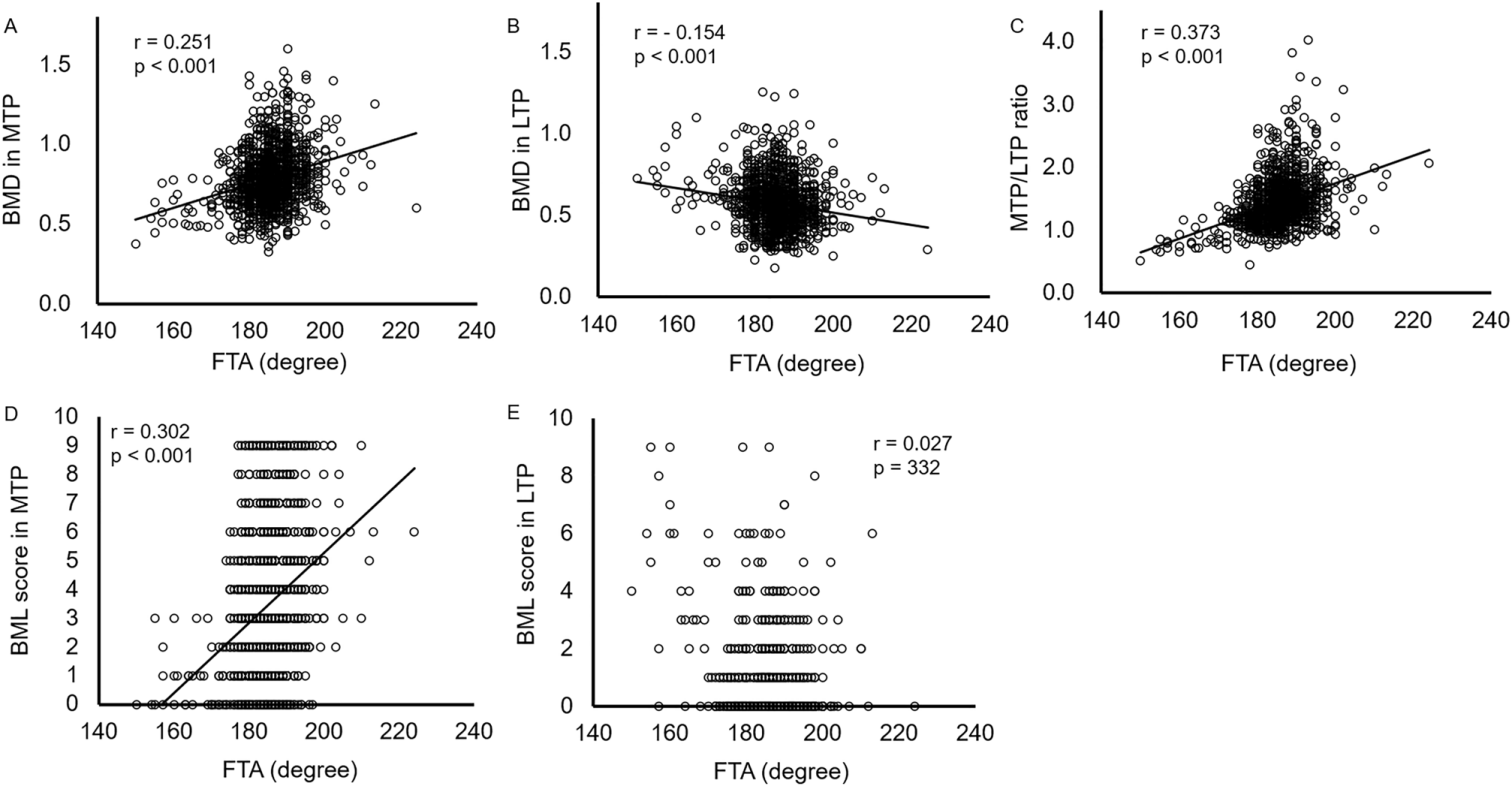
Correlation among medial to lateral balance of bone mineral density (BMD), bone marrow lesion (BML) score, and femorotibial angle (FTA). Scatter plot among BMD or BML score in medial tibia plateau (MTP), lateral tibia plateau (LTP), and MTP/LTP ratio and FTA are shown. Spearman’s correlation coefficients were calculated.

Overall BML scores in the medial femoral condyle, medial tibial plateau, medial femorotibial joint, lateral femoral condyle, lateral tibial plateau, lateral femorotibial joint, patellofemoral joint, and whole joint were 2.0 ± 1.5, 3.5 ± 2.5, 5.5 ± 3.8, 0.5 ± 1.0, 0.5 ± 1.2, 1.0 ± 1.9, 1.1 ± 1.3, and 8.5 ± 5.8, respectively. No significant difference in the BML score of each compartment was found between the sexes (Table 2). The prevalence of BMLs was 90.4% in MTP, 24.2% in LTP, 83.2% in MFC, 28.7% in LFC, 94.0% in medial FT joints, 38.3% in lateral FT joints, and 97.6% in whole joints. While the prevalence of BMLs was higher in the medial compartment than in the lateral compartment, no significant differences were found between men and women in both compartments (Fig. 4). Correlation analysis showed that women had higher BML scores in the MTPs, which were positively and weakly correlated with medial BMD (p < 0.001, r = 0.278) and the MTP/LTP (p < 0.001, r = 0.267) and MTP/metaphysis ratios (p < 0.001, r = 0.243). The MTP/LTP ratio weakly correlated with the FTA (r = 0.373, p < 0.001) (Fig. 5).

Regression analysis showed that high BML scores of the whole joint were positively correlated with loss of knee extension angle (p < 0.001) and BMD in MTP (p < 0.001) and negatively correlated with BMI (p < 0.001), knee flexion angle (< 0.001), BMD in LTP (p = 0.011), and BMD in metaphysis (p = 0.021) (Table 3). Similarly, higher BML scores of the MTP were correlated with lower BMI (p = 0.003), higher loss of knee extension angle (p < 0.001), higher FTA (p < 0.001), higher BMD in MTP (p < 0.001), and lower BMD in LTP (p < 0.001) (Table 3). Contrastingly, the BML scores of the LTP were positively correlated with BMDs of the MTP (p = 0.037) and LTP (p = 0.001) and negatively correlated with BMDs of the metaphysis (p = 0.017) (Table 3).

**Table 3:** Factors affecting bone marrow lesion scores in end-stage osteoarthritis knee.

	Whole joint		MTP		LTP	
	β	P-value	β	P-value	β	P-value
Age	0.017	0.542	− 0.002	0.955	0.042	0.160
Female	− 0.003	0.916	0.019	0.472	0.050	0.984
Body mass index	**− 0.101**	**< 0.001**	**− 0.077**	**0.003**	− 0.054	0.058
Loss of knee extension angle	**0.178**	**< 0.001**	**0.179**	**< 0.001**	0.027	0.354
Knee flexion angle	**− 0.102**	**< 0.001**	− 0.038	0.158	**− 0.112**	**< 0.001**
Femorotibial angle	0.046	0.124	**0.166**	**< 0.001**	**− 0.200**	**< 0.001**
BMD MTP	**0.316**	**< 0.001**	**0.354**	**< 0.001**	**0.076**	**0.037**
BMD LTP	**− 0.087**	**0.011**	**− 0.149**	**< 0.001**	**0.118**	**0.001**
BMD metaphysis	**− 0.080**	**0.021**	− 0.050	0.133	**− 0.088**	**0.017**

Significant values are in [bold].

Linear regression analysis is performed with bone marrow lesion scores in the whole joint, medial tibial plateau (MTP), and lateral tibial plateau (LTP) as dependent variables, and age, females, body mass index, loss of the knee extension angle, knee flexion angle, radiographic femorotibial angle, and bone mineral density (BMD) at the MTP, LTP, and metaphysis were independent variables. β: the standardized regression coefficient.

## Discussion

The present study revealed that higher BML scores in MTP were associated with higher BMD in the MTP in end-stage osteoarthritic knees. This indicated that BMD of the proximal tibia was not low, even in severe osteoarthritic knees with large BMLs. However, a higher BML score in MTP showed a weak correlation with a higher MTP/LTP ratio, indicating medial to lateral imbalance of BMD in the proximal tibia. This information can help determine the surgical strategy or perioperative interventions for bone fragilities.

This study demonstrated detailed age-related changes in BMD of the proximal tibia, which was reduced in women aged > 65 years and not in men. Previous reports have shown that BMD of the proximal tibia was moderately correlated with distal femoral^19,20^, proximal femoral, and lumbar BMDs^20^. Bone strength around the knee joint can be estimated using systemic BMD. However, medial to lateral balance of the bone strength in the proximal tibia was not estimated using systemic BMD; measurement of local BMD in the proximal tibia is more valuable in detecting the medial to lateral balance of the BMD in the proximal tibia.

The MTP/LTP BMD ratio was above 1.0, as previously reported^19,21,22^. A higher MTP/LTP ratio was weakly correlated with higher BML scores, especially in the MTP. This medial to lateral imbalance of the bone strength within the proximal tibia could have been induced by increased sclerotic changes in the medial proximal tibia due to excessive medial loading, leading to severe varus deformity and lateral bone atrophy. Similarly, previous reports have shown that increased medial/lateral BMD ratios of the proximal tibia were positively associated with increased mechanical axis angle and OA worsening^21,23^. Further, Lo et al. reported that the prevalence of medial sclerosis in OA patients is associated with a higher MTP/LTP BMD ratio^22^. In the Framingham study, medial BMLs were correlated with a higher MTP/LTP ratio of BMD^24^. The coronal balance of bone strength within the tibia is an important factor for accurate osteotomy and implantation techniques. Since TKA with mechanical alignment needs balanced load-bearing on the tibial axis, low bone strength at the lateral tibia poses a risk for aseptic loosening^25^.

Higher BML scores in MTP were weakly correlated with higher BMD in MTP. This direct association of BML scores with BMD in MTP will be useful when considering indications for unicompartmental knee arthroplasty (UKA). Bone strength of MTP is one of the major factors that surgeons consider when planning a UKA; periprosthetic fractures of the MTP are frequent and cause severe complications in UKA at a rate of 3.3–7.2%, followed by aseptic loosening of the tibial component^26,27^. Our results suggest that a greater BML score in MTP did not reflect a lower BMD. This information may help determine structural surgical strategy and planning for UKA.

There was a discrepancy in interpreting BML causation between the early- and end-stage knee OA. Mechanical alignments of normal adults originally showed a varus of ≥ 3°, and the mechanical axis was slightly shifted to the medial side^28^. In early-knee OA, excessive contact pressure on the medial FT joint forms BMLs due to meniscus dysfunction or tibial varus^29,30^. We speculated that microcracks caused by excessive loading resulted in bleeding, bone remodeling, and high turnover of bone metabolism in the medial FT joint. In support of this, a previous epidemiological study showed that patients with early-knee OA had low systemic BMD and a high turnover of bone metabolism^16^; with disease progression, repeated bone remodeling caused sclerotic changes in the medial FT joint and accelerated varus deformity. Consequently, BMD in the medial FT joint in end-stage OA knees was not reduced and potentially reflected secondary changes of edema or micro-bleeds within sclerosis. Menopause causes BMD reduction^31^ and modulates subchondral bone remodeling^32^, which possibly caused the positive correlation between BMD and BML scores in women and not in men in our study.

Our results demonstrated that increased BML scores in end-stage osteoarthritic knees did not reflect bone fragility. BML score as an indication of perioperative intervention with a bisphosphonate or parathyroid hormone is controversial. Some reports demonstrated that bisphosphonates improved knee pain in patients with OA^33^ and reduced the need for TKA^34^. Contrastingly, a systematic review concluded that bisphosphonates had no remarkable effect on pain relief and the prevention of OA progression^35^. Ballal et al. also reported no significant benefit of oral bisphosphonates in patients with OA with BMLs in a 12-month clinical trial^36^. Furthermore, among patients with symptomatic knee OA and BMLs, yearly zoledronic acid infusions, compared with a placebo, did not significantly reduce 24-month cartilage volume loss^37^. These reports suggest that bisphosphonate use is not recommended for the treatment of knee OA. Therefore, interventions may not be required to improve bone strength in end-stage osteoarthritic knees with severe BML scores, although they may be necessary for patients with low systemic BMD. Furthermore, longitudinal studies should be conducted to investigate the relationship among the incidence of aseptic loosening or periprosthetic fractures, BMD of the proximal tibia, and BMLs.

This study has several limitations, including its retrospective design. First, the rate of bone metabolism was not evaluated. BMLs reflect bone remodeling; hence, measuring bone metabolic markers would aid in understanding bone quality. Moreover, complications such as renal failure, diabetes, and malignancy should be included in the analysis as they affect bone quality^38^. Second, the clinical outcomes of tibia component migration, aseptic loosening, and periprosthetic fractures were not examined. A longitudinal observational study could reveal the influence of BMLs or BMD on these complications. Third, the associations among knee OA severity, its symptoms, and the presence of BMLs could have been evaluated further. Fourth, the populations of men and women were not balanced, which had a risk for bias in comparisons between men and women. Fifth, the location of ROIs might be affected by the size of the proximal tibia because their size varies based on an individual’s body size. In this study, measurement location was defined clearly and uniformly, although the location might differ among individuals. Lastly, selection bias and reverse causation may have occurred in this retrospective study. Fewer patients with rheumatoid arthritis might lead to selection bias. Also, this study could not conclude whether a lower BML score led to lower BMD by partial weight bearing via pain or lower BMD easily led to microcracks and a higher BML. Further longitudinal analysis is needed to determine the absolute causality. Despite these limitations, this study revealed a positive association between higher BML scores and high BMD in the medial FT joint in women. Further longitudinal studies should investigate its association with clinical outcomes of aseptic loosening and periprosthetic fractures.

## Conclusions

High BML scores in the MTP were positively and weakly associated with high BMD in MTP, which also induced the medial to lateral imbalance of BMD in the proximal tibia. Higher BML scores did not directly reflect focal lower BMD.

## Data Availability

All of data and material are available from the database of Department of Orthopaedic Surgery and Social Medicine, Hirosaki University Graduate School of Medicine.

## Abbreviations

BML: Bone marrow lesions
BMD: Bone marrow density
MTP: Medial tibial plateau
LTP: Lateral tibial plateau
TKA: Total knee arthroplasty
OA: Osteoarthritis
MRI: Magnetic resonance imaging
BMI: Body mass index
ROI: Region of interest
MFC: Medial femoral condyle
LFC: Lateral femoral condyle
FT: Femorotibial
CI: Confidence interval
UKA: Unicompartmental knee arthroplasty
FTA: Femorotibial angle
